# Exome and genome sequencing to unravel the precise breakpoints of partial trisomy 6q and partial Monosomy 2q

**DOI:** 10.1186/s12887-023-04368-5

**Published:** 2023-11-22

**Authors:** Shuang Zhang, Qianwei Cui, Shangying Yang, Fangxia Zhang, Chunxia Li, Xiaoguang Wang, Bo Lei, Xunlun Sheng

**Affiliations:** 1 People’s Hospital of Ningxia Hui Autonomous Region (Ningxia Medical University), Ningxia Eye Hospital, Yinchuan, 750001 China; 2 Gansu Aier Ophthalmology & Optometry Hospital, Lanzhou, 730030 China; 3 Henan Eye Institute, Henan Eye Hospital, People’s Hospital of Zhengzhou University, Henan Provincial People’s Hospital, Zhengzhou, Henan 450003 China

**Keywords:** Structural variations, 2q, 6q, Chromosome translocation, Copy number variation, Partial trisomy 6q, Partial monosomy 2q

## Abstract

**Background:**

Patients with complex phenotypes and a chromosomal translocation are particularly challenging, since several potentially pathogenic mechanisms need to be investigated.

**Case presentation:**

Here, we combined exome and genome sequencing techniques to identify the precise breakpoints of heterozygous microduplications in the 6q25.3-q27 region and microdeletions in the 2q37.1-q37.3 region in a proband. The 5-year-old girl exhibited a severe form of congenital cranial dysinnervation disorder (CCDD) in addition to skeletal dysmorphism anomalies and severe intellectual disability. This is the second case affecting chromosomes 2q and 6q. The individual’s karyotype showed an unbalanced translocation 46,XX,del(2)t(2;6)(q37.1;q25.3), which was inherited from her unaffected father [46,XY,t(2;6)(q37.1;q25.3)]. We also obtained the precise breakpoints of a de novo heterozygous copy number deletion [del(2)(q37.1q37.3)chr2:g.232963568_24305260del] and a copy number duplication [dup(6)(q25.3q27)chr6:g.158730978_170930050dup]. The parental origin of the observed balanced translocation was not clear because the parents declined genetic testing.

**Conclusion:**

Patients with a 2q37 deletion and 6q25.3 duplication may exhibit severe significant neurological and skeletal dysmorphisms, and the utilization of exome and genome sequencing techniques has the potential to unveil the entire translocation of the CNV and the precise breakpoint.

**Supplementary Information:**

The online version contains supplementary material available at 10.1186/s12887-023-04368-5.

## Introduction

Both 6q trisomy and 2q monosomy are rare chromosome abnormalities. Diagnosing genome structural variations (SVs) remains challenging due to the complexity of larger chromosomal events, the complete range of SVs, and the need for appropriate sequencing techniques [1]. A thorough evaluation of all types of SVs could potentially elucidate the diverse clinical manifestations, which may be attributed to deletions of varying sizes in different genes. In this study, we successfully employed a combination of exome and genome sequencing techniques to confirm a diagnosis of pathogenic SV involving an unbalanced translocation between chromosomes 2q and 6q in a family.

The proband initially came to our hospital seeking treatment for ptosis. During the ophthalmic examination, several other concerning features were identified, including downward-slanted palpebral fissures and bilateral optic nerve hypoplasia. Additionally, she exhibited a prominent forehead, midface hypoplasia, hypertelorism, a flat nasal bridge, a short nose, a small mouth with thin lips, micrognathia, and blepharophimosis. She also experienced postnatal growth deficits and developed severe intellectual disability and was affected by significant neurological and skeletal dysmorphism anomalies. This is the second reported case of a patient simultaneously carrying partial monosomy 2q and partial trisomy 6q. In a previous report, a patient carrying the karyotype of 46,XY,der(2)t(2;6)(q37.3;q26) presented intellectual disability, obesity, brachydactyly, and short stature. The author identified the 2q deletion as the primary driver of the phenotype due to AHO-like syndrome (MIM # 600,430) [2]. In comparison with the previous case, our patient presented with a more severe form of congenital cranial dysinnervation disorder (CCDD) (MIM # 620,469), along with abnormal thorax development. This expands the range of phenotypic manifestations associated with the unbalanced translocation between the distal regions of chromosomes 2q and 6q. Her karyotype showed an unbalanced translocation of 46,XX,del(2)t(2;6)(q37.1;q25.3). This variant was transmitted from her unaffected father [46,XY,t(2;6)(q37.1;q25.3)], which led to del(2)(q37.1;37.3) and dup(6)(q25.3;q27). We also obtained the precise breakpoint of a de novo heterozygous copy number deletion [del(2)(q37.1q37.3)chr2:g.232963568_24305260del] and a copy number duplication [dup(6)(q25.3q27)chr6:g.158730978_170930050dup].

The accurate molecular diagnosis of genetic disorders makes it possible to better understand the pathogenesis of complex syndromes, which is imperative to families with a history of diseases and couples with consecutive abnormal fetuses showing similar phenotypes. Identifying pathogenic variants is an excellent method for explaining the correlation between phenotype and genotype and the possible effects on biological mechanisms.

## Materials and methods

### Study participants

Our study conformed to the tenets of the Declaration of Helsinki. Ethical approval for this study was obtained from the ethics committee of the People’s Hospital of Ningxia Hui Autonomous Region, northwest China. Written informed consent was obtained from all participants or their legal guardians before enrollment. Four participants from a family from a nonconsanguineous marriage, including one patient and three unaffected family members, were recruited from the People’s Hospital of Ningxia Hui Autonomous Region. The mother had four pregnancies, two miscarriages and two normal births. The proband was the fourth child, and she had an elder sister with a normal phenotype. The parents denied exposure to toxicants and any adverse personal history. The proband underwent systemic clinical evaluations, including evaluations of medical history and family history and comprehensive ophthalmic examinations. Peripheral blood samples were collected from all participants using 5 mL tubes with ethylene diamine tetraacetic acid (EDTA).

### Whole-exome sequencing (WES)

Genomic DNA was extracted from peripheral blood using a TIANamp Blood DNA kit (DP348-03; Tiangen, Shanghai, China). DNA purity and concentration were measured using a NanoDrop spectrophotometer (Thermo Fisher Scientific, Waltham, Massachusetts). Genome libraries were constructed using the xGen Exome Research Panel v1.0 (Integrated DNA Technologies, Coralville, Iowa) and sequenced on HiSeq 4000 (Illumina, San Diego, CA) to generate 150 bp paired-end reads at a target depth of 100×. The average sequencing coverage was 90-fold, with > 95% of the region of interest covered at least 20-fold. The quality of the raw data was checked by FASTQC (https://www.bioinformatics.babraham.ac.uk/projects/fastqc/). After removing the low-quality reads, the adaptor reads were mapped to the reference genome (GRCh37/hg19) with Burrows‒Wheeler Aligner (BWA). GATK was used for insertion and deletion realignment, quality recalibration, and variant calling. The detected variants were annotated using ANNOVAR. Variants with minor allele frequencies (MAFs) > 0.5% in healthy population frequency databases, including dbSNP, 1000 Genome Project (1000G, http://www.internationalgenome.org/data), the Exome Aggregation Consortium Browser (ExAC, http://exac.broadinstitute.org), and the Genome Aggregation Database (gnomAD, https://gnomad.broadinstitute.org/), were filtered. Novel and rare variants (MAF < 0.5%) were classified and analyzed for pathogenicity according to the American College of Medical Genetics and Genomics (ACMG) guidelines [3].

### Copy number variation sequencing (CNV-seq)

Low-coverage whole-genome sequencing (1× coverage) was used to detect CNVs. Genomic DNA was extracted, followed by random fragmentation and short-read sequencing using the Illumina NextSeq500 sequencer (Illumina, San Diego, CA, USA). The officially recommended Illumina analysis software BclToFastq was used for raw data processing, and clean data were aligned to the human reference genome (GRCh37/hg19) using BWA. CNVkit was applied to detect copy number variations (CNVs) [4, 5]. Candidate regions with values below 0.6 or above 1.4 were considered putative deletions or putative duplications, respectively. Variant calling on CNVs larger than 100 kb was performed using an in-house pipeline, and candidate CNVs were filtered with DGV (http://dgv.tcag.ca/dgv/app/home) and NCBI (https://www.ncbi.nlm.nih.gov/). Decipher (DECIPHER v11.22: Mapping the clinical genome (deciphergenomics.org)), ClinVar (https://www.clinicalgenome.org/), ClinGen (https://www.clinicalgenome.org/), and OMIM (https://www.omim.org/) were included to annotate the genes or genomic regions in the candidate pathogenic CNVs. The annotation information and database frequency were used to perform comprehensive assessments of CNV hazard levels [6].

CNVs were classified and verified through the OMIM, UCSC (http://genome.ucsc.edu/), Database of Genome Variants (DGV), and Decipher databases and divided into five categories (pathogenic, likely pathogenic, uncertain significance, likely benign, and benign).

### Karyotype analysis

High-resolution G-banding karyotype analyses were performed using a standard method. A total of 10 metaphase cells were analyzed. Karyotype summaries were made according to the International System for Human Cytogenetic Nomenclature.

### Oxford nanopore sequencing (ONT)

Genomic DNA isolation was the same as described for WES. DNA was purified with a 1× reaction with Agencourt Ampure XP Beads (NC9959336, Fisher Scientific, Hampton, NH). DNA was then treated with NEB Next Ultra II End-Repair/dA-tailing Module (NEB E7546S, New England Biolabs, Ipswich, Massachusetts) to repair any damaged template DNA. Library preparation was performed according to the manufacturer’s protocol, and sequencing was performed at 48 h on an MK1B MinION. Sequencing and base calling were performed using MinKNOW version 1.1.21 and Metrichor version 1.125 (ONT, Oxford, UK). The native Fast5 files were converted to FASTQ files using Poretools [7].

### Optical genome mapping (OGM)

High-molecular-weight DNA was extracted from whole blood using a Bionano Prep Blood and Cell Culture DNA Isolation Kit (Bionano Genomics #30,033, San Diego, CA, USA). Briefly, blood was subjected to two rounds of red blood cell lysis. After quantitation, white blood cells were immobilized in agarose plugs (Bio-Rad, USA). Plugs were digested with proteinase K (Qiagen, Germany) and washed with wash buffer (Bionano Genomics) and TE buffer (Thermo Fisher Scientific, USA). DNA was recovered, dialyzed, homogenized, and quantitated. DNA labeling was processed with a Bionano Prep DLS DNA Labeling kit (Bionano Genomics #30,071) according to the kit protocol. In brief, DNA was labeled with DLGreen fluorophores using DLE-1 enzyme at 37 °C for 2 h, digested with proteinase K at 50 °C for 30 min, and cleaned up with 1× DLE-1 buffer. Subsequently, the DNA backbone was stained with DNA stain, 5× DTT, and 4× flow buffer for 1 h and homogenized overnight at 4 °C. Labeled DNA was loaded on a Saphyr chip (Bionano Genomics) and run on a Saphyr instrument (Bionano Genomics). De novo genome map assembly was performed using Bionano Solve version 3.4 (Bionano Genomics), and structural variations (SVs) (based on assembled maps) and CNVs (based on molecular coverage) were called against the human reference genome (GRCh38/hg38). Data were analyzed with Bionano Access and Bionano Tools on Saphyr Compute Servers (Bionano Genomics) [8, 9].

### Targeted next-generation sequencing (NGS) and sanger sequencing

Targeted NGS was performed to validate the sequences of the SVs acquired by Bionano Optical Mapping. Polymerase chain reaction (PCR) amplification regions and primers were designed according to the breakpoint regions of SVs (Table 1). PCR was carried out using a LongAmp Taq PCR Kit (E5200S; NEB, Ipswish, MA). A total of 50–100 ng of gDNA was used as the PCR template (conditions: 96 °C for 5 min followed by 35 cycles of 96 °C for 30 s, 60 °C for 30 s, and 72 °C for 4 min, with a final extension at 72 °C for 4 min). PCR product purification, library construction, sequencing, and data analysis were the same as described for WES. Sanger sequencing was used to validate the breakpoint junctions. Primer sequences and coordinates are listed in Table 1.

**Table 1:** Primers used for targeted NGS and Sanger sequencing

Amplified region	Length	Sequence of oligonucleotide(5’-3’)
**chr2:232957822–232,969,822**	12,000 bp	F: CAGTCTTTCTTCTCCACTCCCACCCA R: CAGCCATCACAGCCACAGTTCAGG
**chr6:158724792–158,736,792**	12,000 bp	F: TTCTGTCACTTCCTCCCTTTGCCTTCA R: CGCCTGCTACCCTACACCTTACCAACA
**chr2:232963168–232,963,968**	800 bp	F: AATGCAGCTCTTTGATGGAACA R: TTCTACTGGCCTGTGACTTACTTG
**chr6:158730578–158,731,378**	800 bp	F: CGGGCCAGGTAACCTCATG R: CAGGGTGGTGCTGTTGTGC

## Results

### Clinical characteristics

**Fig. 1:**
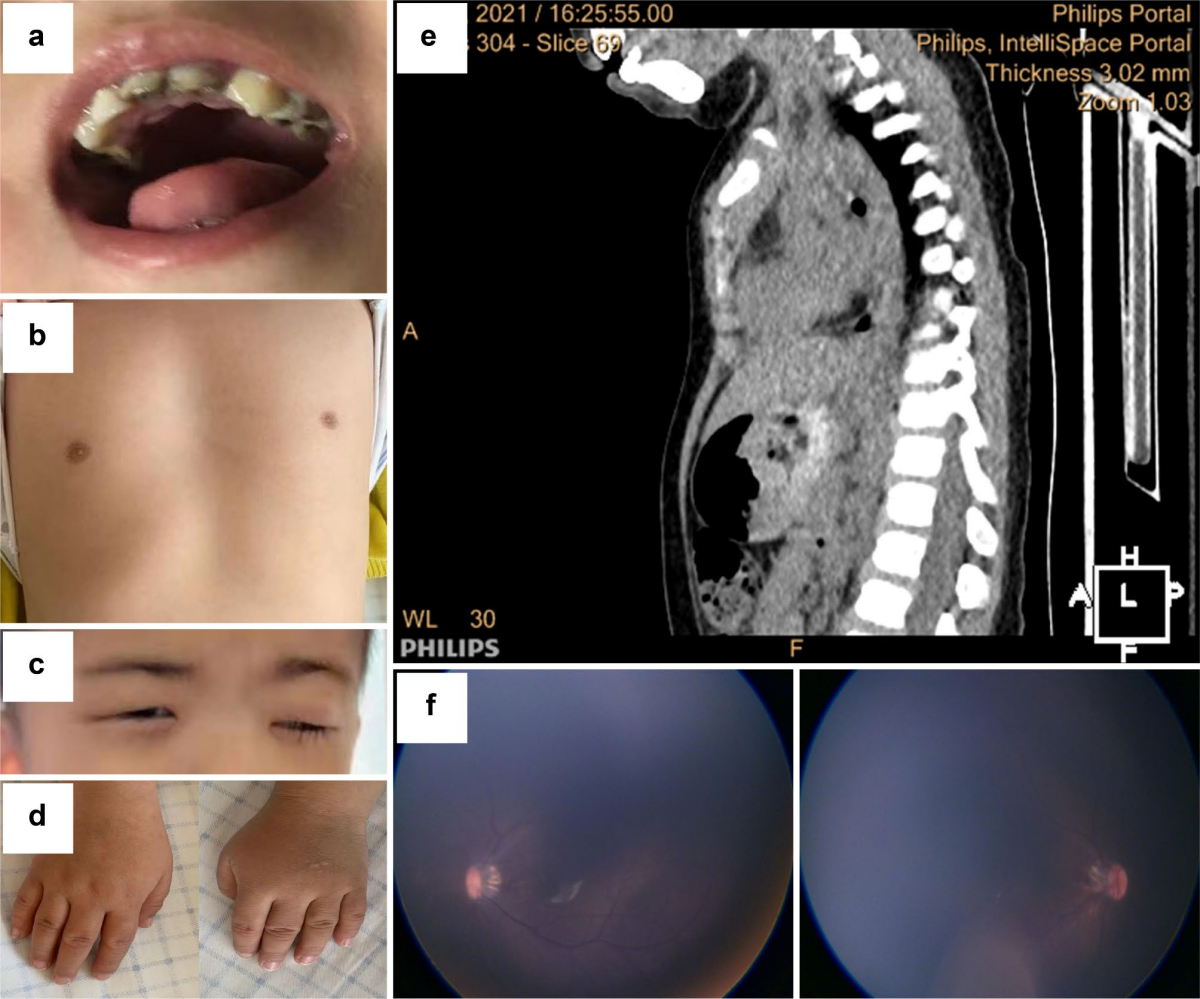
Characteristics of phenotypes. **(a)**. The proband has deciduous teeth, showing bad enamel development with severe caries (7 teeth). **(b)** The proband shows malformations of the thorax and short ribs. **(c)** The proband has downward-slanted palpebral fissures and accompanying lower eyelid entropion and trichiasis. The eyelashes are longer on the right eye than on the left eye. **(d)** The proband has small hands, the fingers of which are short and contracted. **(e)** CT shows that the narrow thorax and the sternum’s lower half deform inwardly. **(f)** The proband shows bilateral optic nerve hypoplasia from the examination screening wide-field digital retina imaging system (Panocam)

The 5-year-old proband was first brought to our eye hospital to be evaluated for “bilateral ptosis”. After the comprehensive ophthalmic examination, we found that the girl had terrible visual acuity (which could not be tested reliably because of intellectual disability). She had severe congenital CCDD with downward-slanted palpebral fissures resulting in a false appearance of ptosis, with the left palpebral fissure being 2 mm and the right fissure being 1 mm, accompanied by lower eyelid entropion and trichiasis. The length of both eyelashes was different (longer for the right eye than for the left eye) (Fig. 1c). Slit-lamp examination showed clear corneas, regular anterior chambers and clear lenses. Both pupils were equally sized and reacted to direct and consensual stimulation. Intraocular pressures were normal. She showed severely delayed development, including bilateral optic nerve hypoplasia from the screening wide-field digital retina imaging system (Panocam) examination (Fig. 1f).

The girl was the fourth child of this family and was delivered without complications in the 38th week of gestation. No specific reason for the two preceding abortions is known. The patient weighed 2950 g (between the 10th and 25th percentile), was 50 cm tall (between the 50th and 75th percentile), had a biparietal diameter of 9.4 cm (between the 25th and 50th percentile), and had an occipitofrontal diameter of 30 cm (between the 75th and 90th percentile) at birth [10]. The girl’s nonconsanguineous parents and her elder sister (10 years old) were all healthy. She had facial deformities, including a prominent forehead, midface retraction, hypertelorism, a flat nasal bridge, a short nose, a small mouth with thin lips, micrognathia, and blepharophimosis. She exhibited a short neck with a low nuchal hairline and abnormal deciduous teeth with bad enamel development and severe caries (7 teeth). Most tooth surfaces were covered with debris (Fig. 1a). Although the body size at birth was within the normal range, it gradually fell behind the mean value of Chinese children.

In the latest examinations at 5 years old, the girl showed severely delayed development of clinical features. She weighed 17.8 kg (between the 25th and 50th percentile) and was 104 cm tall (between the 3rd and 10th percentile) [10]. She had a bone disorder associated with the developmental anomaly of the skeleton and the teeth. Bone age studies (X-ray of the hand) indicated retarded bone development with a skeletal age of approximately four years. The long bones were shortened. The upper limbs, especially the arms, were shortened, with a functional arm span 95 cm in length. Her hands and feet were small, 9 cm for the right-hand length and 10 cm for the left-hand length. The fingers on both hands were short and contracted (Fig. 1d). Her chest circumference was 45 cm, and malformations of the thorax and short ribs were observed. The thorax was narrow, and the lower half of the sternum deformed inwardly, which was the result of abnormal and unbalanced growth of costal cartilage connecting each of the ribs and sternum (Fig. 1b and e). Consistent with the narrow thorax, she had recurrent pneumonia many times.

Neurological examination showed abnormal motor skills and coordination. Myotonus was regular, and tendon reflexes were present. She exhibited severe intellectual disability; her ability to learn to walk was delayed until she was nearly 3 years and 8 months old. The Denver Developmental Screening Test showed severe delays in gross motor skills and language. Serologic tests and urine analysis, including amino acid screening assays, were performed regularly. Urine sediments showed no sign of cytomegalic inclusion cysts. Serological tests for syphilis, hepatitis, mumps, herpes simplex, cytomegalovirus, mycoplasma, and toxoplasma were unremarkable. Endocrinological evaluation of thyroid hormones, including thyroid-stimulating hormones T3, T4 and GH, showed typical results.

The proband’s uncle, the son of her father’s aunt, showed a similar phenotype. He had facial deformities with a prominent forehead, midface retraction, hypertelorism, a flat nasal bridge, esotropia, and a small mouth with thin lips. He was also found to have malformations of the thorax and short ribs and died of pneumonia at 35 years old.

### Two copy number variations were found in the proband

**Fig. 2:**
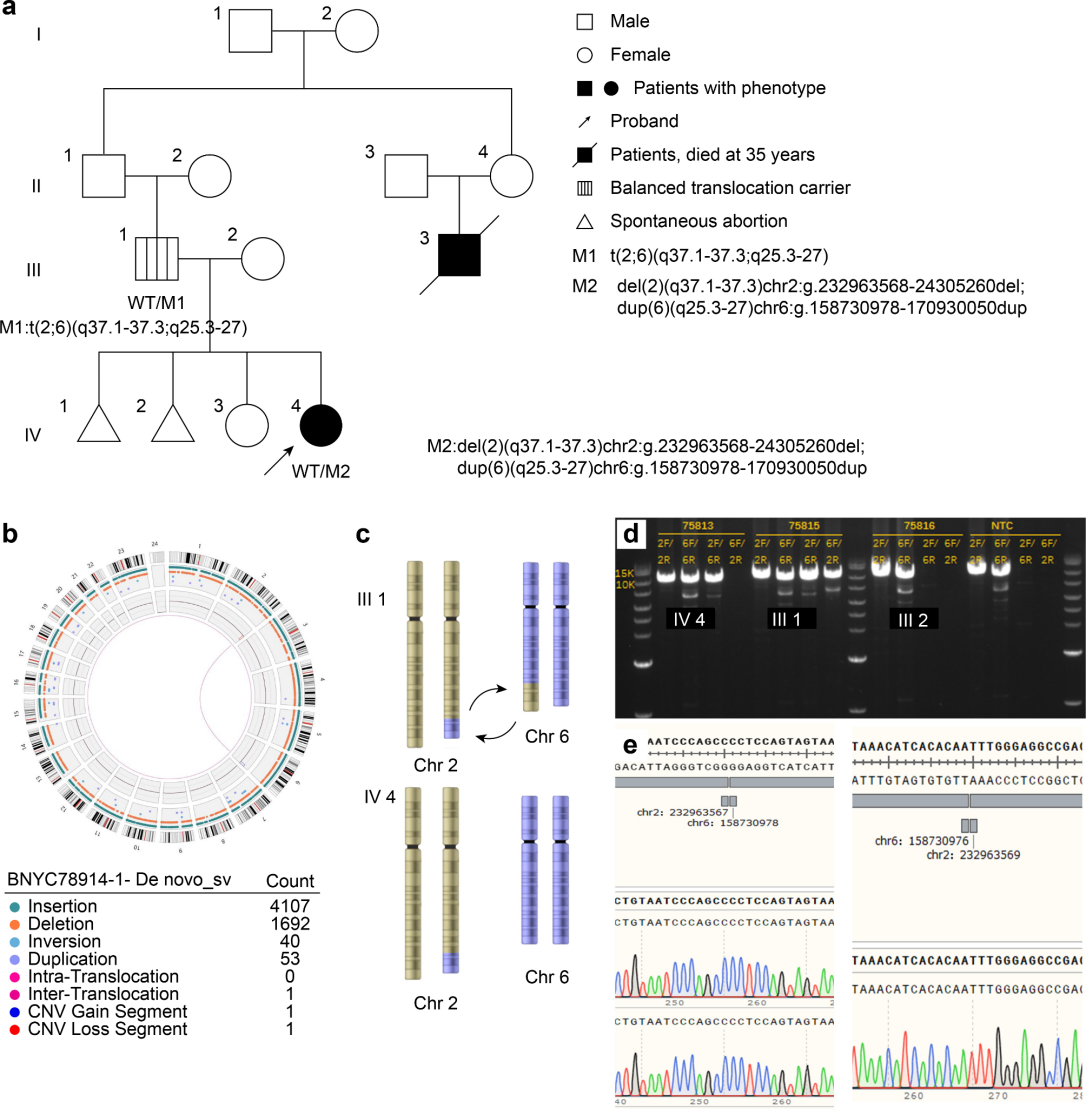
Pedigree information and Sanger verifying results [32]. **(a)** In this pedigree, WT indicates a normal allele. M1 represents the balanced translocation t(2;6)(q37.1-37.3;q25.3-27). M2 represents the unbalanced translocation del(2)(q37.1-37.3)chr2:g.232963568-24305260del and dup(6)(q25.3-27)chr6:g.158730978-170930050dup. Proband (IV4) is indicated by the arrow. **(b)** Circle map showing the balanced translocation between chromosome 2 and chromosome 6. **(c)** III1 shows the balanced translocation between chromosome 2 and chromosome 6 of the proband’s father. IV4 shows the unbalanced translocation between chromosome 2 and chromosome 6 of the proband, with 2q monosomy and 6q trisomy. **(d)** PCR products including the chromosome rearrangement site of the breakpoint. The proband (IV4) shows three bands at 1000 bp, the proband’s father (III1) shows four bands at 1000 bp, and the mother (III2) and the normal control show two bands without a rearrangement breakpoint. **(e)** Sanger sequencing identifying the chromosome rearrangement breakpoint: chr2:232963567/chr6:158730978 and chr6:158730976/chr2:232963569

To investigate the potential pathogenic variants of the proband, WES was initially performed on the proband’s family, including her father, mother, elder sister, and herself. Only reads filtered through quality control standards were preserved for further genetic analysis. Then, genetic variants in the exonic and splicing regions with minor allele frequencies (MAFs) ≥ 0.001 in the gnomAD database (http://www.gnomad-sg.org/) were excluded. InDels and nonsynonymous variants predicted to be deleterious by multiple bioinformatic tools were selected as candidate pathogenic variants. No significant variants were identified based on the standards and guidelines of the American College of Medical Genetics and Genomics, the inheritance pattern of the variants and the clinical phenotypes of the proband; surprisingly, two copy CNVs (https://www.deciphergenomics.org/), a heterozygous deletion in chromosome 2 and a heterozygous duplication in chromosome 6, were found in the proband, while none of her family members carried these two CNVs (Fig. 2a III2, III1). Then, we applied CNV-seq to identify the pathogenic CNVs; CNV-seq analysis revealed a 12.01 Mbp duplication in the 6q25.3-q27 region, involving 53 protein-coding genes (49 OMIM genes), and a 9.23 Mbp deletion in the 2q37.1-q37.3 region, involving 85 protein-coding genes (74 OMIM genes) (Fig. 2a IV4), which are listed in the Supplementary Table. There were 17 OMIM morbid genes in the deleted region and 19 in the duplicate region, which are listed in Table 4. Because two chromosomes were involved, we presumed that these CNVs resulted from translocation, which was proven by Sanger sequencing. Thus, the de novo heterozygous copy number deletion, del(2)(q37.1q37.3)chr2:g.232963568_24305260del, and copy number duplication, dup(6)(q25.3q27)chr6:g.158730978_170930050dup, were identified.

**Fig. 3:**
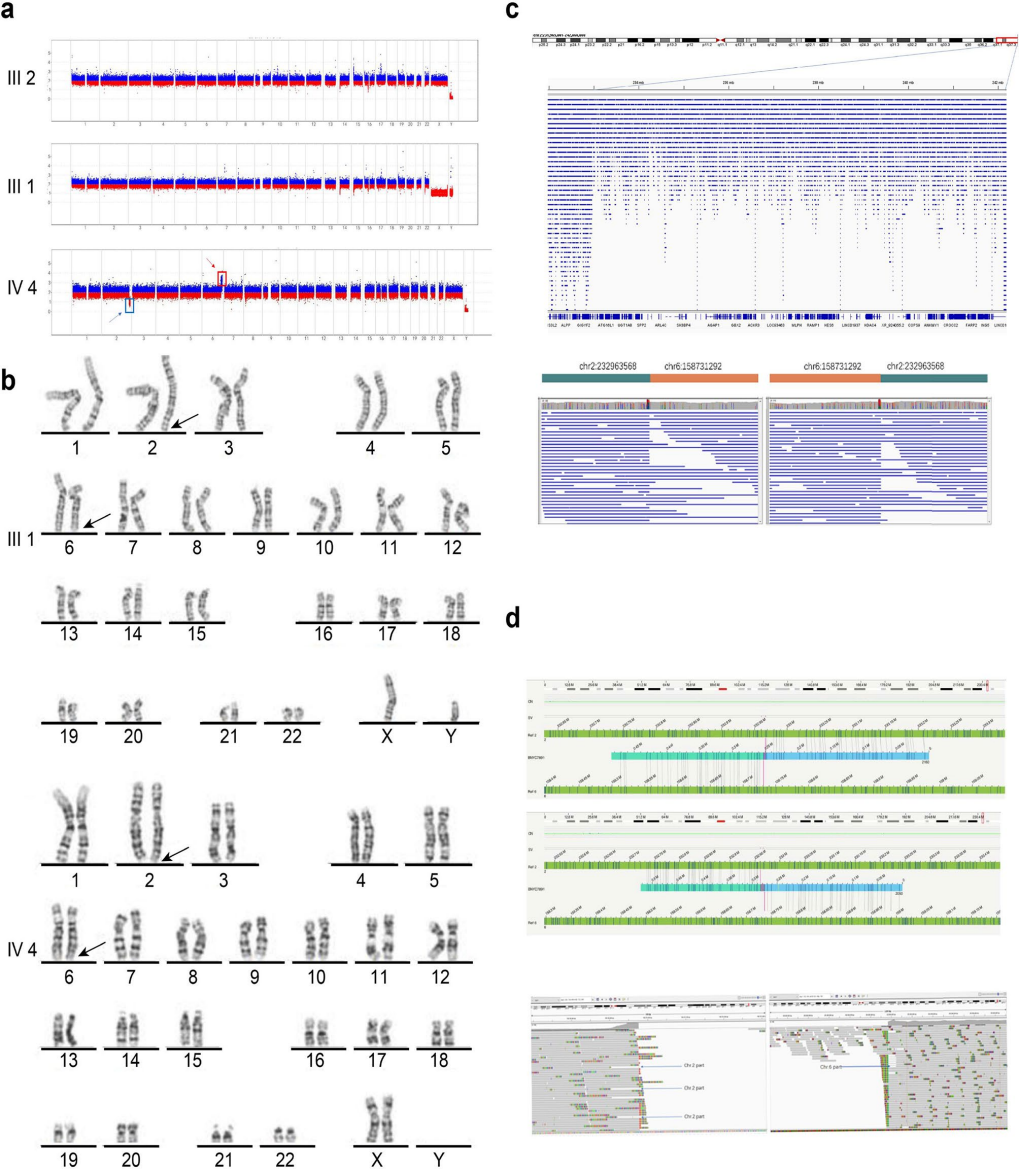
The sequencing results of CNV-seq, karyotype, ONT, and OGM. **(a)** The proband’s mother (III2) and father (III1) showed no copy anomalies on CNV-seq. The proband (IV4) exhibits a heterozygous deletion in chromosome 2 and a heterozygous duplication in chromosome 6; the red arrow shows copy number duplication, and the blue arrow shows copy number deletion. **(b)** Karyotype shows the chromosome translocation between 2q and 6q of the proband’s father (III1) and the proband (IV4). **(c)** Long-read technology (ONT) shows that the reads decrease aberrantly at 2q37.1-37.3, and it provides information about the chromosome rearrangement at chr2:232963568/chr6:158731292. **(d)** OGM shows two translocation breakpoints, one on chromosome 2 and one on chromosome 6

The CNV-seq results were consistent with the WES results, which failed to cover all CNVs. Furthermore, ONT and OGM also confirmed the read loss at 2q37.1-37.3 and the heterozygous recombination of Chr2:233963568 and Chr6:158731292 (hg38), which was not reported in gnomAD (Fig. 3c and d).

### Balanced translocation was identified in the proband’s father

Because the CNVs occurred at the end of chromosomes 2q37 and 6q25-27, we presumed that they were created by translocation derived from her parents. However, CNV-seq could not directly identify the balanced translocation t(2;6), which was derived from her father. Next, we tried other long-read techniques to ensure the precise site and possible origin by ONT and OGM. Both techniques could accurately detect the site of unbalanced and balanced translocations. Karyotyping was also used to detect translocation. The proband’s karyotype was 46,XX,del(2)t(2;6)(q37.1;q25.3) (Fig. 3b IV4), and her father’s karyotype was 46,XY,t(2;6)(q37;q25.3) (Fig. 3b III1). We assumed that the father carried a balanced translocation because he had typical healthy male characteristics. Chromosome 2 of the proband had its end replaced by bands from chromosome 6, resulting in one copy of 2q37.1-37.3 and three copies of 6q25.3-27. The proband’s mother had a regular 46, XX female karyotype. The diagram of the inheritance pattern of translocated chromosomes 2 and 6 from the father to the proband is shown in Fig. 2a and b, and 2c. The balanced translocation breakpoints of the father could not be found by CNV-seq. The father has a balanced reciprocal translocation between 2q37 and 6q associated with a normal phenotype. During the rearrangement, one bp was missing, with “G” occurring in the noncoding GCC area of the *NGEF* gene in chromosome 2q and “T” missing in the noncoding area of the *SYTL3* gene in chromosome 6q.

The risk of unbalanced translocation in their next child is estimated at 16 out of 18, while the risk of balanced translocation is estimated at 1 out of 18. There is also a 1 out of 18 possibility for the child to be normal [11].

### Exact breakpoints of the balanced translocation were revealed

The translocation breakpoint regions were narrowed down to approximately 10 kb intervals based on the positions of the fluorescent probes (GRCh38/hg38) (Table 2).

**Table 2:** Exact breakpoints of the balanced translocation

Chromosome	Breakpoint region	Region length (bp)
2	Chr2:232955038–232,966,616	11,578
	Chr6:158730918–158,742,900	11,982
6	Chr6:158725206–158,736,951	11,745
	Chr2:232960564–232,968,828	8264

In light of the previous results, long PCR was performed smoothly (Fig. 2d). Targeted next-generation sequencing of long PCR products revealed the exact breakpoints of the balanced translocation. Sanger sequencing finally showed the two adhesions of chromosomes 2 and 6 (Fig. 2e). The proband showed a *de novo* heterozygous copy number deletion, del(2)(q37.1q37.3)chr2:g.232963568_24305260del, and a copy number duplication, dup(6)(q25.3q27)chr6:g.158730978_170930050dup, which was derived from parental balanced translocation [t(2;6)(q37.1-37.3;q25.3-27)].

## Discussion

Here, we report a proband with characteristic features of the clinical syndrome, including downward-slanting palpebral fissures; delayed development of bilateral optic nerve hypoplasia; facial deformity with a small mouth, thin lips, micrognathia, and blepharophimosis; short neck; abnormal deciduous teeth; and malformations of the thorax and short ribs. The proband showed severe CCDD and mental and developmental disability. To our knowledge, this is the second report that depicts the features of partial 6q trisomy and partial monosomy 2q with translocation. The duplicated region in our patient, 6q25.3-q27, includes 53 protein-coding genes (49 OMIM genes), and the deleted region, 2q37.1-q37.3, includes 85 protein-coding genes (74 OMIM genes).

In a previous report, the proband was described as carrying 46,XY,der(2)t(2;6)(q37.3;q26), along with a balanced translocation [t(2;6)] in his father and sister [2]. This chromosomal abnormality is associated with AHO-like syndrome, also known as brachydactyly mental retardation syndrome (BDMR syndrome). This syndrome is characterized by several features, including brachydactyly type E (48%), overweight and obesity (34%), cognitive-behavioral issues (79%), and dysmorphic craniofacial or skeletal dysmorphism (86%) [12]. The main contributing factor to the proband’s phenotype was believed to be monosomy of the 2q37.3-qter region. Patients with chromosome 2q37 deletion syndrome exhibit a wide range of clinical manifestations, which can be attributed to the varying length and deletion of specific genes in this region. Among the OMIM morbid genes, several genes have been identified as candidates correlated with the phenotype of our proband’s 2q37 deletion. *COL6A3* (MIM *120,250), which encodes type IV collagen, has been associated with short stature, obesity, and brachymetaphalangia in affected individuals [13]. *PDCD1* (MIM *600,244), which codes for a cell surface membrane protein, has been reported to be associated with malformation, as it plays a role in programmed cell death or apoptosis in embryogenesis [14]. The *HDAC4* (MIM *605,314) gene, which is involved in chromosomal packaging, has been implicated in the major phenotypes of BDMR syndrome through its ability to repress transcription [12].

An imbalance in gene dosage can have deleterious effects, with deletions typically causing a more severe clinical presentation than duplications [15]. In addition to BDMR, our proband also showed severe CCDD, which manifested as downward-slanting palpebral fissures and delayed development of bilateral optic nerve hypoplasia. These symptoms indicate central nervous system abnormalities and ocular defects, suggesting a partial role of trisomy 6q. Both trisomy 6q and monosomy 2q are rare chromosome abnormalities. Trisomy 6q is associated with severe physical and intellectual disability, hypertelorism, feeding difficulties, and dysmorphic features (microcephaly, acrocephaly, prominent forehead, flat nasal bridge, downward-slanting palpebral fissures, carp mouth, micrognathia, short webbed neck, club feet, and flexion deformity) [16, 17]. While both trisomy 6q and monosomy 2q37 commonly exhibit signs of psychomotor retardation, growth retardation, thin upper lip, prominent forehead, flat nasal bridge, micrognathia, and deformities of hand and foot (Table 3), trisomy 6q appears to be more associated with severe neurological abnormities, whereas monosomy 2q is more linked to skeletal abnormities along with mild neurological abnormities. Among the genes in the duplicated region of our proband, the *DLL1* (MIM *606,582) gene is involved in neurodevelopmental disorders with nonspecific brain abnormalities in the trisomy 6q region. The *DLL1* gene encodes a Notch ligand and is essential for developing the nervous system and somites [18]. Additionally, the *PSMB1* (MIM *602,017) gene is responsible for the breakdown of intracellular proteins, and its malfunction can lead to neurodevelopmental disorders characterized by microcephaly, hypotonia, and absent language [19]. Our proband presented with severe skeletal dysmorphism abnormalities that are more closely associated with monosomy 2q. It appears that both monosomy 2q and trisomy 6q contribute to the complex phenotype of our proband. Partial duplication of the distal long arm of chromosome 6 alone can result in a severe phenotype [16]. This is commonly accompanied by severe intellectual disability, hallmark facial malformation, short stature, and severe motor abnormalities. Compared with pure monosomy, the phenotypic difference was believed to be due to a larger trisomic region exerting the effect of deletion when deletion and duplication occurred in the same patient.

The accurate molecular diagnosis of genetic disorders makes it possible to better understand the pathogenesis of complex syndromes, which is imperative to families with a history of diseases and couples with consecutive abnormal fetuses showing similar phenotypes. It is necessary to accurately predict the features (copy, content, and structure) of SV discovery and genotyping. However, it remains a challenge to find a single method or technology that can comprehensively detect all SVs within a genome [1]. A single method can neither provide a definite determination of the breakpoint nor reveal the location and orientation. The combination of exome and genome sequencing techniques is the optimum selection to detect the precise site of SVs. Exome and genome sequencing are proposed to be applied to pediatric patients with congenital anomalies or intellectual disabilities [20]. The genome sequencing approach is a complementary method to locate the accurate site of the SVs when the WES technique and the phenotype point to a pathological CNV as the leading cause. Thus, exome sequencing rapidly located the pathological CNVs, even though the CNV information is obscure. OGM and ONT can efficiently complement exome sequencing to locate the breakpoint of SVs, identify the leading cause and have the potential to uncover more complex genomic structures that are missed by low-resolution methods. Although the long-read technique takes advantage of other techniques in detecting SVs [1], it is unlikely that, in our case, these variants could be identified from long-read data alone without prior knowledge of the region of interest.

In the present study, we highlight the role of combining exome and genome sequencing techniques in resolving the SVs with a translocation between 2q and 6q in one family. In this study, we initially utilized WES to identify the pathological CNVs on chromosomes 2q and 6q but failed to detect the precise location. WES is a commonly used, high-throughput short-read technology for detecting exact breakpoints and has resulted in dramatic increases in novel gene discovery for Mendelian disorders. It initially used a hybridization microarray approach and has been successful in identifying regions and distinguishing alleles with high sensitivity. Nevertheless, WES has limitations in capturing larger genomic variants (CNVs, ≥ 50 bp) and cannot detect SVs reliably due to the technical and analytical challenges around identifying and interpreting SVs that are associated with deletions or duplications and could be overlooked throughout the full scope of the genome. Additionally, breakpoints located within repetitive regions of the genome are unmappable by WES, which means a lower sensitivity. In addition, balanced translocations cannot be detected since they do not alter chromosome copy numbers. Therefore, not surprisingly, WES only provided results that pointed to a pathological CNV with a deletion at the terminal part of 2q37 and a duplication of 6q that may be the leading cause, but without precise resolution of breakpoints. Therefore, we tried other genome sequencing methods.

With the development of next-generation sequencing, ChIP-Seq allows for the sequencing of DNA fragments rather than hybridizing them to an array, making it possible to develop a robust statistical model that describes the complete analysis procedure and allows the computation of essential confidence values for the detection of CNVs. This has led to CNV sequencing as a promising technique. Low-resolution CNV-seq is a short-read whole-genome sequencing method that has proven particularly powerful in detecting SVs, variants in GC-rich regions, and variants in noncoding regulatory regions [21]. We further used CNV-seq to identify the pathogenic CNVs; CNV-seq rapidly identified the existence of the pathogenic CNV and classified the abnormality as a 12.01 Mbp copy number duplication of distal 6q25.3-q27 and 9.32 Mbp copy number deletion of distal 2q37.1- q37.3. However, there are short-read technical challenges in resolving the exact structures of SVs given their substantial diversity and proximity to repetitive regions [22]. This method could locate the sequence but could not detect the existence of the translocation.

When considering the two involved chromosomes, we applied the long-read technique to identify the precise site and possible origin. The long-read genome sequencing methods OGM and ONT also made it possible to map the breakpoint precisely to the region covered by 2q and 6q [23]. Based on nanochannel-based genome mapping technology, OGM utilizes enzymes to label high-molecular-weight DNA without breaking the DNA or polymerase chain reaction (PCR), and fluorescently tagged mega-base size DNA is obtained. These long DNA fragments are linearized through pillars and imaged in nanochannels as a long stretch of single-stranded DNA that passes through a protein nanopore that is stabilized in an electrically resistant polymer membrane [24]. By applying a voltage across this membrane, sensors detect the ionic current changes caused by nucleotides occupying the pore in real time as the DNA molecule passes through. Although it cannot offer base-level sequence information, it can visualize DNA structure and reveal a wide spectrum of SVs. Nanopore sequencing is another technique that directly detects the input molecule without DNA amplification or synthesis; there is no apparent limit to the length of DNA that can be sequenced [25]. Long-read techniques have the advantage of reads of 10–100 kb, allowing for more accurate mapping, particularly over repetitive regions, and facilitating phasing [26]. We obtained the precise breakpoint of a de novo heterozygous copy number deletion [del(2)(q37.1q37.3)chr2:g.232963568_24305260del] and a copy number duplication [dup(6)(q25.3q27)chr6:g.158730978_170930050dup], and the parental balanced translocation was apparent. However, unlike the limits of CNV-seq in balanced translocation, long reads can locate the causative region of balanced and unbalanced translocations, but CNV-seq is much more cost-effective. A combination of karyotyping and CNV-seq is another suggested approach for the diagnosis of submicroscopic unbalanced genomic rearrangements [27]. Although karyotyping is a routine standard cytogenetic method to detect SVs, its drawbacks are low resolution and the inability to indicate the genomic localization and orientation of duplicated segments or insertions (CNV microarrays). It usually takes much longer to obtain sequencing information. Therefore, the combination of exome and genome sequencing is a more efficient choice.

This study typified a pathological CNV offspring with unbalanced translocation caused by parental balanced translocation. Approximately one in every 300–500 individuals has a balanced reciprocal translocation according to varied estimates [28]. Balanced translocations can take the form of inversions or translocations and lead to CNVs, which often involve changes in DNA dosage. CNVs, partial chromosomal deletions and duplications are significant contributors to the genome variability among individuals and can either be pathogenic or have no clinical consequences. A diverse set of rearrangements involving the exchange of segments between chromosomes is common in humans. Most balanced translocation carriers have no phenotypic consequences but have a higher risk of infertility, miscarriage, and unbalanced progeny. Moreover, their offspring may have a continuous spectrum of phenotypic effects of pathogenic CNVs, from adaptive traits to underlying causes of disease to embryonic lethality risk of an associated reciprocal translocation [29]. Translocations have also been productive in identifying new candidate genes underlying common clinical phenotypes that may arise from dysfunction of any number of genes, as in alveolar soft-part sarcoma [30], acute myeloid leukemia [31], and autism [32]. Researchers are struggling to identify a rapid, cost-effective solution with a low per-base error rate to assess this issue. This solution could provide new information to study translocation formation and may suggest ways to prevent its occurrence. Thus, exome and genome sequencing techniques could be applied to resolve chromosomal balanced translocation and complex SVs. This combination ensures the accurate determination of variants. Considering the cost, time, and throughput, the combination of WES and CNV-seq may be a good alternative for SV detection but cannot directly detect balanced translocations; thus, other combinations should be taken into consideration.

In conclusion, the combination of exome and genome sequencing techniques is suggested to secure precise breakpoints of the location of SVs, which could be in the form of CNVs or translocations, and provide evidence of an irregular phenotype due to the translocation between 6q + and 2q-. The genome sequencing techniques could be CNV-seq, ONT, or OGM, with CNV-seq being much more cost-effective than the other two techniques. Our study also emphasizes that accurate molecular diagnosis of genetic disorders is critical to interpreting the pathogenesis of complex syndromes and supplying evidence of the formation of replication-based mechanisms for complex structural variations, which is imperative to families with a history of diseases and couples with consecutive abnormal cases showing similar phenotypes. It is also helpful for the patient’s mother to undergo prenatal examination in the event of future pregnancies.

**Table 3:** Comparison of the phenotype between the 2q- and 6q + syndromes

Phenotype	Del 2q	Dup 6q	Present case	[2]t(2;6)(q37.3;q26)
Psychomotor retardation	+	+	+	+
Growth retardation	+	+	+	
Microcephaly		+		
Broad/round face	+			
Prominent forehead	+	+		+
Flat nasal bridge	+	+	+	
Downward-slanting palpebral fissures		+	+	
Short palpebral fissures	+			
High arched palate		+		+
Carp/bow shaped mouth/Microstomia		+	+	
Thin upper lip	+	+	+	+
Micrognathia	+	+	+	
Short webbed neck		+	+	+
Joint contractures		+	+	
Camptodactyly	+	+	+	
Finger ulnar deviation/syndactyly	+	+		
Small feet	+	+	+	+
Clubfoot		+		
Obesity	+			+

**Table 4:** The genes in the CNVs with OMIM numbers

CNV	MIM gene	Phenotype MIM number	Gene MIM number	Phenotype
chr2:g.232963568_24305260del	AGXT	#259900	*604285	Hyperoxaluria, primary, type 1
	ATG16L1	#611081	*610767	Inflammatory bowel disease (Crohn disease) 10
	CAPN10	#601283	*605286	Diabetes mellitus, noninsulin-dependent 1
	COL6A3	#158810	*120250	Bethlem myopathy 1
		#616411	*120250	Dystonia 27
		#254090	*120250	Ullrich congenital muscular dystrophy 1
	D2HGDH	#600721	*609186	D-2-hydroxyglutaric aciduria
	KIF1A	#614255	*601255	NESCAV syndrome
		#614213	*601255	Neuropathy, hereditary sensory, type IIC
		#610357	*601255	Spastic paraplegia 30, autosomal dominant /recessive
	MLPH	#609227	*606526	Griscelli syndrome, type 3
	NDUFA10	#618243	*603835	Mitochondrial complex I deficiency, nuclear type 22
	PDCD1	#126200	*600244	Multiple sclerosis, disease progression, modifier of
		#605218	*600244	Systemic lupus erythematosus, susceptibility to, 2
	PER2	#604348	*603426	?Advanced sleep phase syndrome, familial, 1
	SAG	#258100	*181031	Oguchi disease-1
		#613758	*181031	Retinitis pigmentosa 47, autosomal recessive
		#620228	*181031	Retinitis pigmentosa 96, autosomal dominant
	TRAF3IP1	#616629	*607380	Senior-Loken syndrome 9
	TWIST2	#200110	*607556	Ablepharon-macrostomia syndrome
		#209885	*607556	Barber-Say syndrome
		#227260	*607556	Focal facial dermal dysplasia 3, Setleis type
	UGT1A1	#218800	*191740	Crigler-Najjar syndrome, type I
		#606785	*191740	Crigler-Najjar syndrome, type II
		#237900	*191740	Hyperbilirubinemia, familial transient neonatal
		#601816	*191740	Bilirubin, serum level of, QTL1
		#143500	*191740	Gilbert syndrome
	DTYMK	#619847	*188345	Neurodegeneration, childhood-onset, with progressive microcephaly
	HDAC4	#619797	*605314	Neurodevelopmental disorder with central hypotonia and dysmorphic facies
	ACKR3	#619215	*610376	?Oculomotor-abducens synkinesis
chr6:g.158730978_170930050dup	DLL1	#618709	*606582	Neurodevelopmental disorder with nonspecific brain abnormalities and with or without seizures
	ERMARD	# 615544	615532	?Periventricular nodular heterotopia 6
	IGF2R	#114550	*147280	Hepatocellular carcinoma, somatic
	LPA	#618807	*618807	LPA deficiency, congenital; Coronary artery disease, susceptibility to
	MPC1	#614741	*614738	Mitochondrial pyruvate carrier deficiency
	PDE10A	# 616921	*610652	Dyskinesia, limb and orofacial, infantile-onset
		#616922	*610652	Striatal degeneration, autosomal dominant
	PLG	#619360	*173350	Angioedema, hereditary, 4
		#217090	*173350	Plasminogen deficiency, type I ;Dysplasminogenemia
	PRKN	#211980	*602544	Adenocarcinoma of lung, somatic
		#167000	*602544	Ovarian cancer, somatic
		#600116	*602544	Parkinson disease, juvenile, type 2
	RNASET2	# 612951	*612944	Leukoencephalopathy, cystic, without megalencephaly
	RSPH3	#616481	*615876	Ciliary dyskinesia, primary, 32
	SMOC2	#125400	*607223	Dentin dysplasia, type I, with microdontia and misshapen teeth
	SOD2	#612634	*147460	Microvascular complications of diabetes 6
	TBP	#607136	*600075	Spinocerebellar ataxia 17
		#168600	*600075	Parkinson disease, susceptibility to
	TBXT	#615709	*601397	Sacral agenesis with vertebral anomalies
		#182940	*601397	Neural tube defects, susceptibility to
	THBS2	#603932	*603932	Lumbar disc herniation, susceptibility to
	ACAT2	614055	*100678	?ACAT2 deficiency
	PNLDC1	#619528	*619529	Spermatogenic failure 57
	CEP43	*605392		Myeloproliferative disorder
	PSMB1	#620038	*602017	?Neurodevelopmental disorder with microcephaly, hypotonia, and absent language

### Electronic supplementary material

Below is the link to the electronic supplementary material.

Supplementary Material 1

Supplementary Material 2

## Data Availability

The sequencing data is available on https://ngdc.cncb.ac.cn/gsa-human/browse/HRA005556, and the accession number is PRJCA019803.
