# Supplementary material for: Exome and genome sequencing to unravel the precise breakpoints of partial trisomy 6q and partial Monosomy 2q
Source: BMC Pediatr. 2023 Nov 22;23:586. doi: 10.1186/s12887-023-04368-5 (PMC10664609; doi:10.1186/s12887-023-04368-5)
Supplement: Supplementary file 2 — Supplementary Material 2 [file 12887_2023_4368_MOESM2_ESM.docx]

Related  figures


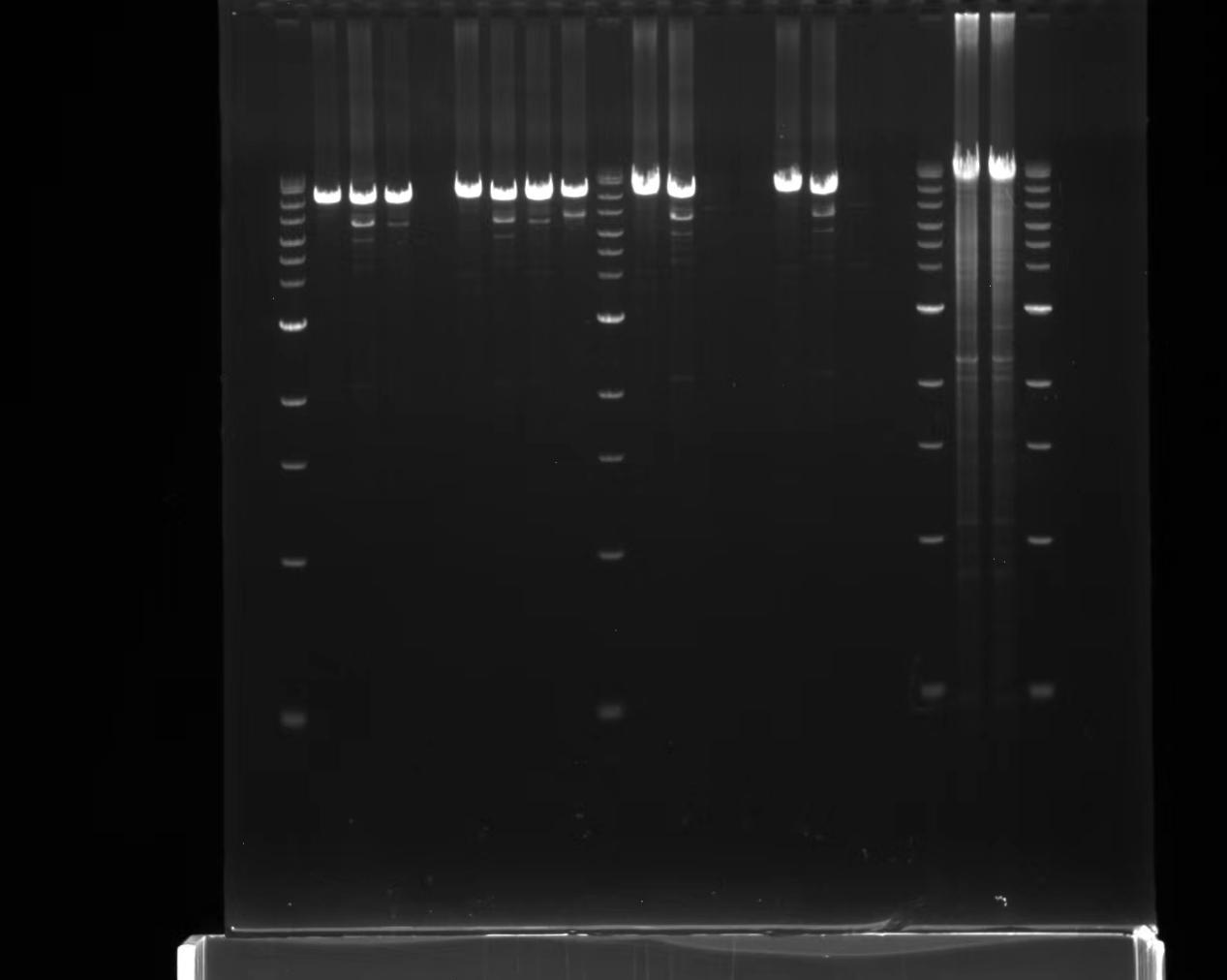


Figure R1. The original figure of figure 2d, PCR products including the chromosome rearrangement site of the breakpoint.


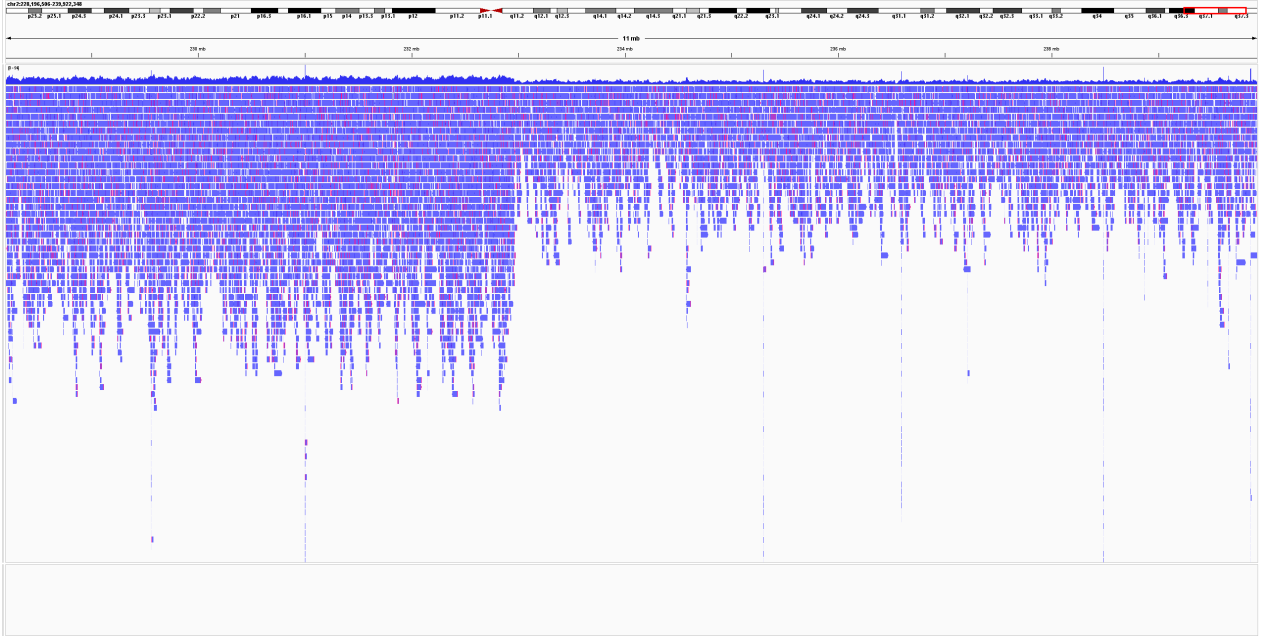


Figure R2. The enlargement of figure 3c, ONT shows the reads decrease aberrantly at 2q37.1-37.3.
